# Two-year changes in sleep duration are associated with changes in psychological distress in adolescent girls and boys: the fit futures study

**DOI:** 10.1080/21642850.2022.2147936

**Published:** 2022-11-21

**Authors:** Jonas Linkas, Luai Awad Ahmed, Gabor Csifcsak, Nina Emaus, Anne-Sofie Furberg, Gunn Pettersen, Kamilla Rognmo, Tore Christoffersen

**Affiliations:** a Department of Health and Care Sciences, UiT The Arctic University of Norway, Narvik, Norway; b College of Medicine and Health Sciences, Institute of Public Health, United Arab Emirates University, Al Ain, UAE; c Department of Psychology, UiT The Arctic University of Norway, Tromsø, Norway; d Department of Health and Care Sciences, UiT The Arctic University of Norway, Tromsø, Norway; e Faculty of Health and Care Sciences, Molde University College, Molde, Norway; f School of Sport Sciences, UiT The Arctic University of Norway, Alta, Norway; g Department of Research and Development, Finnmark Hospital Trust, Alta, Norway

**Keywords:** Psychological distress, sleep duration, depressive symptoms, anxiety symptoms, adolescence

## Abstract

**Objective:**

Studies indicate an inverse association between sleep duration and psychological distress. We aimed to explore associations between changes in sleep duration and changes in psychological distress in girls and boys.

**Methods:**

The Fit Futures Study is a broad adolescent study providing data from 373 girls and 294 boys aged 15–18 years collected in 2010/2011 (FF1) and 2012/2013 (FF2). Psychological distress was measured by the Hopkins Symptom Checklist (HSCL-10) and sleep duration was self-reported. Change score variables were calculated as the change between baseline and follow-up for sleep duration and HSCL-10, respectively. Associations between changes in sleep duration and changes in HSCL-10 were explored by linear regressions, in gender-stratified analyses.

**Results:**

At FF1, girls and boys slept on average 6.93 (SD = 1.08) and 7.05 (SD = 1.20) hours per night respectively, and correspondingly, 6.83 (SD = 1.19) and 6.85 (SD = 1.21) at FF2. At FF1, 22.8% of the girls and 25.8% of the boys slept ≤ 6 h per night, and correspondingly 28.0% and 28.2% at FF2. In girls and boys, one unit increase (30 min) in sleep duration was associated with a decrease in HSCL-10 score of *B* [95% CI] = −0.090 [−0.131, −0.048], *p *< 0.001, and −0.054 [−0.091, −0.017], *p* < 0.001, respectively. The associations remained significant after adjusting for confounders.

**Conclusion:**

Our findings show that increased sleep duration was associated with decreased psychological distress during adolescence. Future studies should examine the causality between sleep duration and psychological distress.

## Introduction

Depressive disorders and anxiety disorders increase dramatically during adolescence (Costello et al., 2003), especially among girls (Nolen-Hoeksema & Girgus, 1994; Xie et al., 2021). Subclinical symptoms of anxiety and depression are even more prevalent in this age group (Bakken, 2019). Such symptoms are often termed ‘psychological distress', defined as ‘a state of emotional suffering characterized by symptoms of depression and anxiety' (Mirowsky & Ross, 2002). Self-reported levels of psychological distress in Norway were recently reported to be 31% and 12% among girls and boys, respectively (Bakken, 2019). Adolescents with psychological distress are at risk of developing mental disorders (Silva et al., 2020). Therefore, longitudinal studies exploring whether factors are associated with psychological distress during adolescence are central for the development of effective prevention measures.

According to a review of the sleep-deprived human brain, sleep loss has been associated with negative emotional processing, including irritability, emotional volatility, and anxiety (Krause et al., 2017). Neuroimaging studies have shown that these emotional changes are associated with increased activity in the amygdala and with a decreased connection between amygdala and the prefrontal cortex (Krause et al., 2017). The change in brain activity indicates that short sleep duration hampers the prefrontal cortex’s ability to execute regulatory control of emotions (Krause et al., 2017). Sleep loss seems to reduce the restoration of central noradrenergic signaling that occurs during a full night of sleep (Krause et al., 2017), and in this respect, it might play a pivotal role in mood and anxiety disorders (Brunello et al., 2003). Interestingly, sleep loss has been shown to reduce positive mood more than to increase negative mood, and anhedonia has been associated with reductions in non-rapid eye movement (NREM) slow-wave sleep (Finan et al., 2015; Finan et al., 2017). A sufficient amount of rapid eye movement (REM) sleep has been linked with the overnight dissipation of emotional sensitivity, and too little REM sleep has been associated with increased activity in areas in the limbic brain involved in negative mood (Ben Simon et al., 2020). Furthermore, sleep loss has been associated with a negative emotional memory dominance (Walker & van Der Helm, 2009). This memory bias may in the long run contribute to depression (Walker & van Der Helm, 2009). Additionally, inadequate sleep has been shown to lead to higher-order-emotional-dysfunction, including interpersonal conflict, social withdrawal and loneliness, (Ben Simon et al., 2020), which in turn have been associated with depression and anxiety (Beutel et al., 2017). A meta-analysis on prospective associations between short or long sleep and depression in adults, support the mechanistic linkage between sleep duration and clinical depression (Zhai et al., 2015).

The American Academy of Sleep Medicine and the National Sleep Foundations recommend that young people aged 13–18 years should sleep 8–10 h every night (Hirshkowitz et al., 2015; Paruthi et al., 2016). However, most adolescents do not meet this recommendation (Carskadon, 2011). In a large study on Norwegian adolescents aged 16–18 years, mean sleep duration on weekdays was 6 h and 25 min (Hysing et al., 2013). This finding is consistent with a report concluding that adolescent sleep loss and sleepiness are serious public health issues (Touitou, 2013). A meta-analysis including adolescents 12–20 years of age found that sleep disturbances are more common among adolescents with depressive disorders compared to age-matched healthy controls. Furthermore, the same study claimed that sleep disturbance was more likely to be prospectively associated with depression rather than in the opposite direction (Lovato & Gradisar, 2014). In healthy adolescents, a systematic review and meta-analysis concluded that sleep duration was both cross-sectionally and prospectively associated with mood (depressed mood, anxiety, anger, negative affect and positive affect) (Short et al., 2020).

A cross-sectional study on Swedish adolescents aged 13–18 years reported a negative correlation between sleep duration and psychological distress (Mazzer et al., 2019). Similarly, a study on 419 youths 15 years of age from the US found associations between both short and long sleep duration and psychological distress the following day (Fuligni et al., 2019). In support, longer sleep duration in adolescents, measured by sleep diary was associated with lower negative affect the next day (Shen et al., 2022). In contrast, no association between sleep duration and psychological distress was reported in a study on 12-year-olds (Jamieson et al., 2020). Based on this inconsistency, more prospective studies to investigate age-related changes in sleep and psychological distress are warranted (Jamieson et al., 2020). Indeed, there are indications that sleep duration at the age of 15 is prospectively associated with both anxiety and depression symptoms as long as six years later (Orchard et al., 2020). In addition, the association between insomnia and depressive symptoms has been reported to be stronger in girls than in boys (Langvik et al., 2019), suggesting that investigations on the association between sleep duration and psychological distress should be gender stratified.

Overall, there are inconsistent findings on prospective associations between sleep duration and psychological distress in healthy adolescents. More knowledge about such associations is central for the prevention of psychological distress. Previous findings suggest that such examinations should be gender stratified. The aim of this study is to explore associations between change in sleep duration and change in psychological distress in adolescent girls and boys.

## Materials and methods

### Study population and design

**Figure 1.**
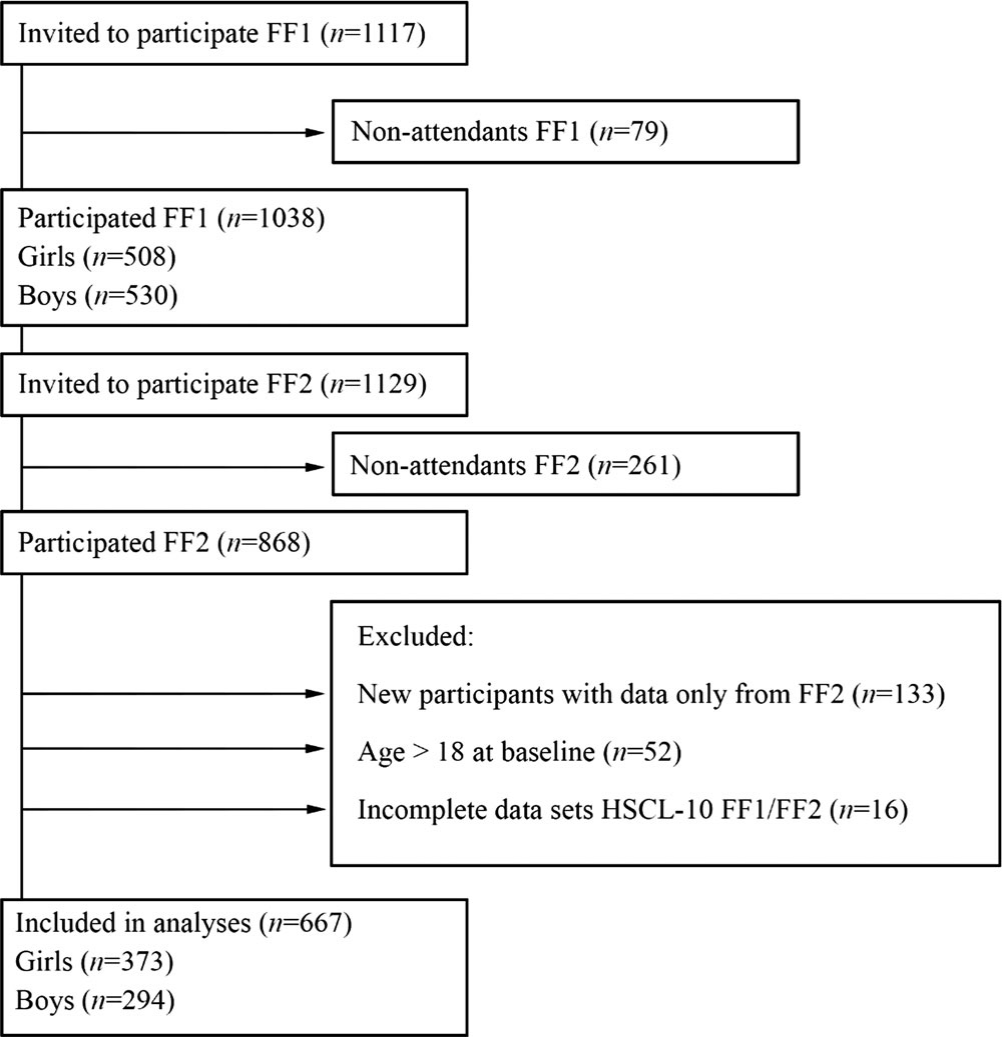
Study flowchart, Fit Futures (FF1) 2010–2011 and Fit Futures 2 (FF2) 2012–2013.

All first-year upper secondary school students from two municipalities in Northern Norway in 2010–2011 were invited to join the Fit Futures Study (FF1), a comprehensive health study. Two years later, in 2012–2013, a follow-up study was conducted (FF2). Further details about Fit Futures are available elsewhere (Winther et al., 2014). Data were collected at the Clinical Research Unit, the University Hospital of North Norway (UNN) in Tromsø. At FF1, 1117 students were invited to participate, of which 1038 (92.9%) attended (FF1). At FF2, all participants that attended FF1 and all third-year upper secondary school students were invited, where a total of 868 attended. After the exclusion of participants aged 19 years or older at baseline, our final sample consisted of 667 participants with complete data on the outcome variable, Hopkins Symptom Check List (HSCL-10) at both FF1 and FF2 (Figure 1). Participants lost to follow-up did not differ on baseline data for neither sleep duration nor HSCL-10 items (data not shown).

All participants provided written informed consent. For participants below 16 years of age, written informed consent was also provided from a parent/guardian. Our study was conducted according to the Declaration of Helsinki. The Norwegian Data Protection Authority (reference number 2009/1282) and The Regional Committee of Medical and Health Research Ethics (reference number: 2019/60811/REK Nord) approved the study.

### Measurements and questionnaires

A web-based general questionnaire was used to collect data about lifestyle, health, and disease. Clinical examinations, collection of blood samples and interviews (on the use of contraceptives, presence of acute or chronic diseases) were conducted by qualified research nurses.

#### Outcome: Hopkins symptom checklist (HSCL-10)

Psychological distress was measured with HSCL-10, which was included in the web-based questionnaire at both time points. HSCL-10 has been reported to have high reliability and validity (Strand et al., 2003). The scale contains six items measuring symptoms of depression and four items measuring symptoms of anxiety during the last week (Strand et al., 2003). The severity of symptoms is reported as ‘none' (1), ‘slightly’ (2), ‘much' (3), and ‘very much' (4). Cronbach’s alpha for girls was 0.87 and 0.93 at baseline and follow-up, and correspondingly 0.82 and 0.87 for boys. The individual score of the 10 items was calculated for baseline and follow-up, respectively, and used in cross-sectional models. A change score variable (FF2-FF1) was created and used as an outcome variable in the prospective models. There were 373 girls and 294 boys with complete data on HSCL-10 at both time-points.

The item ‘Have you experienced sleeplessness during the last week?’ was included in HSCL-10. To ensure robust findings, we did supplementary analyses excluding this item.

#### Main exposure variable: sleep duration

Sleep duration was measured with one question in the web-based questionnaire: ‘How many hours sleep do you normally get per night?' at both time-points. The lowest category was ‘four hours or less'. The successive categories increased 0.5 h per category (‘4.5 h', ‘5 h’, ‘5.5 h', etc.), except for the highest category that was ‘12 h or more'. The lowest category was coded as 4 h, and the highest as 12 h.

These numerical variables were used in cross-sectional models. A categorical variable was created to describe short and long sleep duration at baseline and follow, up respectively. The three categories were: ‘≤6 h', ‘>6–9 h' and ‘>9 h'. A change score variable for sleep duration (FF2-FF1) was calculated and served as exposure in the prospective models. There were 372 girls and 291 boys with data on sleep duration at both time-points.

#### Potential confounders

Based on the literature, we assessed potential confounders (Linkas et al., 2022a; Zhai et al., 2015). For variables with data from both time-points, we assessed the change scores (FF2-FF1).

High school program was collected from the school administration system with the alternatives: ‘general studies', ‘sports and physical' and ‘vocational'.

##### Lifestyle and health variables from questionnaire

At baseline, smoking and snuff use were reported as ‘no, never' (1), ‘sometimes’ (2) and ‘daily' (3). At follow-up, smoking and snuff use were reported as ‘no, never' (1), ‘in the past, but not now' (2) ‘sometimes' (3) and ‘never’ (4), and ‘no, never' (1) and ‘in the past, but not now' (2) were collapsed into one category: ‘no, never' (1). Description of coding and categories of alcohol intake, physical activity in four categories, menarche, Pubertal Development Scale (PDS) for boys and self-rated health are described elsewhere (Linkas et al., 2022a).

##### Variables on health and medication from clinical measurements and interview

Height and weight were measured without shoes and in light clothing at both time points. An automatic electronic scale, The Jenix DS 102 stadiometer (Dong Sahn Jenix, Seoul, Korea) was used to measure weight. Body Mass Index (BMI) was calculated as weight (kg) divided by height in meters squared (m^2^).

Description of coding and categories of chronic disease, current infection and use of contraceptives are described elsewhere (Linkas et al., 2022a).

##### Biomarker variables from laboratory measurements

Information about the measurement of Vitamin D (25(OH)D), High-sensitive C-reactive protein (hs-CRP) and inflammatory markers are described elsewhere (Cashman et al., 2016; Grimnes et al., 2010; Linkas et al., 2022a; Schistad et al., 2020).

### Statistical analysis

The statistical analyses were conducted with the Statistical Package of Social Science (SPSS v. 28). We chose a significance level of *p *< 0.05 as an indication of statistical significance. We conducted residual analyses to assess linearity, distribution, variance homogeneity and to explore outliers. We tested exposure variables and potential confounders for multicollinearity. Since girls have been shown to exhibit higher levels of psychological distress than boys in adolescence (Bakken, 2019) and because the female gender is an independent risk factor for insomnia (Hayley et al., 2015), we did all analyses separately for girls and boys.

We conducted independent samples t-tests to compare sleep duration in girls and boys. This was done at both baseline and follow-up. We conducted paired samples t-tests to compare sleep duration at baseline and follow-up, stratified on gender. Baseline characteristics are presented for three categories of sleep duration and gender-stratified, with means and standard deviations for continuous variables, and proportions for categorical variables. One-way ANOVA was used to compare continuous demographic data across the three categories of sleep duration. When Levene’s test indicated unequal variances, Welch’s corrected *F*- and *p*-values are reported. Fisher’s exact test was used to compare categorical data across sleep duration.

Linear regressions were conducted to estimate the unstandardized beta regression coefficients and 95% confidence intervals (CI) between sleep duration and HSCL-10 (outcome variable), cross-sectionally, at baseline and follow-up separately. To explore prospective associations, similar linear regressions were conducted to estimate the effect of change in sleep duration on change in HSCL-10. Crude associations between the change score of sleep duration and change score of HSCL-10 were estimated (Model 1). Potential confounders were tested in simple regressions: For variables with data from both time-points, we assessed the change scores (FF2-FF1), and for variables without data from FF2, we assessed variables from FF1. We adjusted for change scores of the potential confounders because they are not stationary, but rather fluctuate over time. Confounders were included in multivariable regression analysis (Model 2) when the *p*-value was below 0.10. Missing values were accounted for by listwise deletion.

As supplementary analyses, we did the same cross-sectional and the prospective models without the sleep item in HSCL-10: ‘Have you experienced sleeplessness during the last week?’. Finally, we did supplementary analyses adjusting for baseline values of potential confounders, to explore whether the association between changes in sleep duration and changes in psychological distress was prospectively associated with baseline confounders.

### Ethics statement

All participants provided written informed consent. Participants below 16 years additionally provided written informed consent from a parent/guardian. The study was conducted in accordance with the Declaration of Helsinki and was approved by the Norwegian Data Protection Authority (reference number 2009/1282). The Regional Committee of Medical and Health Research Ethics has also approved the study (reference number 2011/1702/REK Nord), and the present project (reference number: 2019/60811/REK Nord).

## Results

### Description of sleep duration and psychological distress

**Figure 2.**
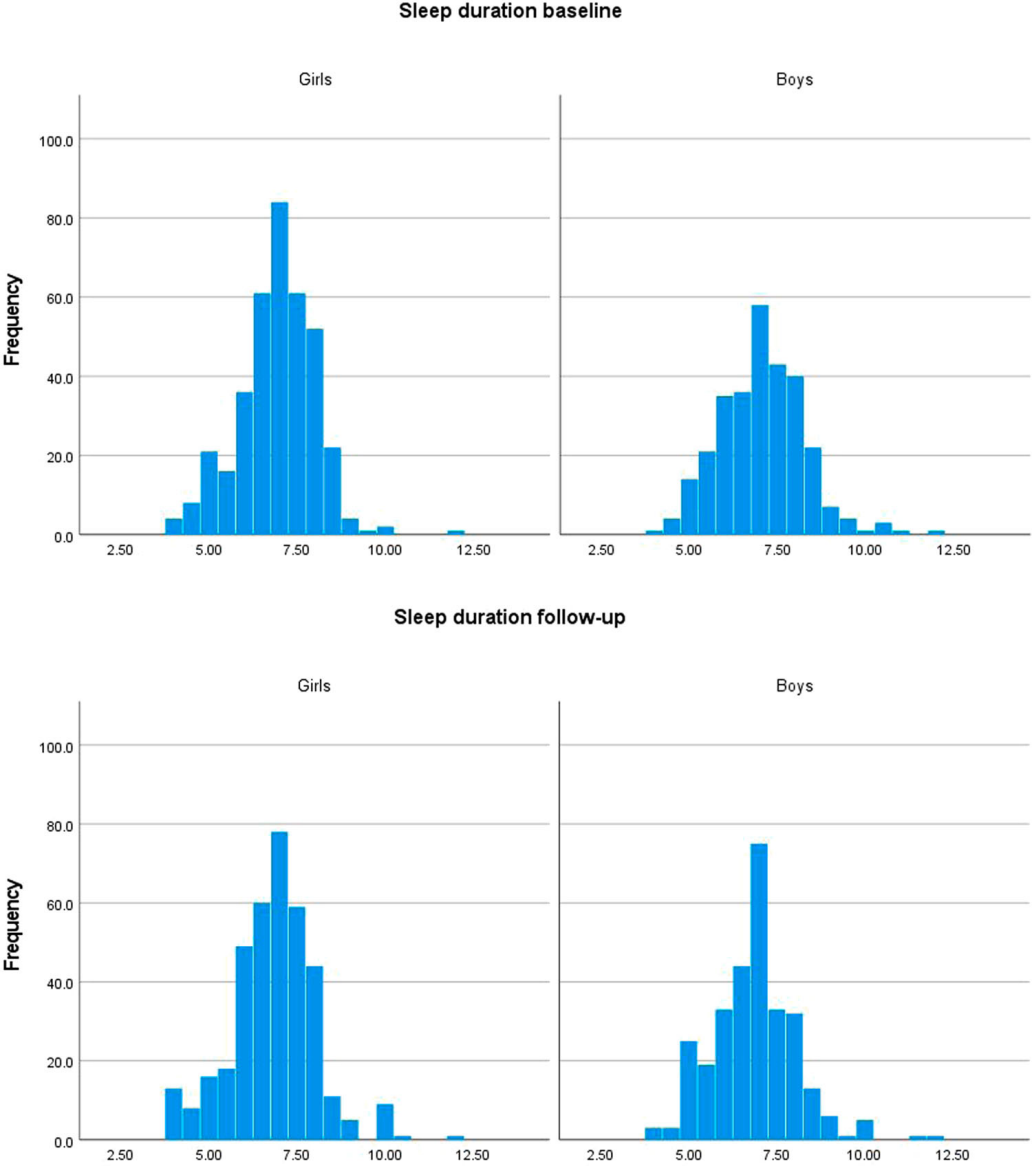
Sleep duration in hours at baseline and follow-up, in girls and boys, respectively. Fit Futures 2010–2011 and 2012–2013.

In both genders, sleep duration was close to normally distributed at both time-points (Figure 2). At baseline, 22.8% of the girls and 25.8% of the boys reported sleep duration of ≤6 h. At follow-up, the corresponding percentages were 28.0% and 28.2%. At baseline, 1.1% of the girls and 3.4% of the boys reported sleep duration of >9 h. At follow-up, the corresponding percentages were 3.0% and 2.7%, respectively. In girls, the mean (SD) sleep duration of 6.93 (1.08) hours at baseline was not significantly different from the mean sleep duration of 6.83 (1.21) hours at follow-up, *p* = 0.170. Boys reported a mean (SD) sleep duration of 7.05 (1.20) hours at baseline and 6.85 (1.19) hours at follow-up, a decrease from baseline to follow-up which was statistically significant, *p* = 0.007. Sleep duration among girls was not significantly different from sleep duration among boys at baseline, *p* = 0.154. Nor was there a gender-difference in sleep duration at follow-up, *p* = 0.839.

Mean (SD) of HSCL-10 increased significantly from baseline to follow-up for both genders. In girls, the increase was from 1.59 (0.56) to 1.69 (0.64), and, in boys, from 1.36 (0.41) to 1.41 (0.48). These means correspond to HSCL-10 means in Norwegian adolescents where 26.9% of the girls and 10.8% of the boys were above the clinical cut-off of 1.85 (Linkas et al., 2022b).

### Baseline characteristics

In girls, HSCL-10 scores differed across categories of sleep duration. The highest level was seen in those who slept ≤6 h. Further, girls that slept ≤6 h smoked more, were less physically active and drank more alcohol compared to girls that slept longer. Also, in boys, those who slept ≤6 h had higher levels of HSCL-10 compared to those who slept longer. A higher proportion of boys who slept ≤6 h smoked, snuffed, and drank alcohol compared to those who slept longer. A higher proportion of boys that attended a vocational high school slept ≤6 h compared to boys that attended general studies (Table 1).

**Table 1.** Baseline characteristics for girls and boys, stratified on sleep duration. Fit Futures 2010–2011.

	Girls							Boys						
	≤6 h		>6 h to >9 h		≥9 h			≤6 h		>6 h to >9 h		≥9 h		
	*n*	Mean (SD)	*n*	Mean (SD)	*n*	Mean (SD)	*p*-value*	*n*	Mean (SD)	*n*	Mean (SD)	*n*	Mean (SD)	*p*-value*
Age (years)	85	16.11 (0.38)	280	16.16 (0.44)	8	16.13 (0.84)	0.513	75	16.17 (0.55)	199	16.08 (0.48)	17	16.12 (0.78)	0.414
Self-rated health	85	3.58 (0.84)	277	4.05 (0.71)	8	3.50 (0.54)	0.087	74	3.96 (0.86)	199	4.24 (0.86)	17	3.94 (0.90)	0.210
HSCL-10	85	1.86 (0.69)	280	1.51 (0.48)	8	1.71 (0.67)	**<0**.**001**	75	1.50 (0.48)	199	1.31 (0.36)	17	1.32 (0.47)	**0**.**014**
Body height (cm)	85	1.64 (6.22)	279	165.24 (6.73)	8	165.59 (7.34)	0.463	75	176.49 (6.85)	199	177.69 (6.26)	17	179.36 (6.46)	0.184
Body weight (kg)	85	59.56 (11.21)	279	60.75 (9.99)	8	62.95 (21.08)	0.659	75	70.99 (16.36)	199	69.50 (13.10)	17	74.98 (16.64)	0.271
BMI (kg/m**^2^**)	85	22.13 (4.38)	279	22.24 (3.38)	8	22.82 (6.90)	0.948	75	22.73 (4.68)	199	21.97 (3.71)	17	23.28 (5.02)	0.214
Age menarche (years)	85	12.58 (1.31)	274	12.73 (1.08)	8	12.88 (0.99)	0.501							
hs-CRP (mg/ L)	69	1.88 (3.00)	253	1.41 (3.24)	8	1.07 (1.44)	0.512	64	1.72 (4.60)	183	1.25 (2.37)	16	1.33 (1.46)	0.740
IL-6 (NPX)	69	3.08 (9.67)	254	2.79 (0.49)	8	2.80 (0.49)	0.086	68	2.80 (0.50)	191	2.88 (0.67)	17	2.54 (0.55)	0.082
TGF – α (NPX)	69	3.94 (0.54)	254	3.88 (0.60)	8	3.98 (0.70)	0.711	68	3.64 (0.62)	191	3.57 (0.57)	17	3.40 (0.44)	0.281
TRANCE (NPX)	69	5.52 (0.63)	254	5.50 (0.62)	8	5.81 (0.35)	0.351	68	5.99 (0.52)	191	6.02 (0.51)	17	6.05 (0.61)	0.901
TWEAK (NPX)	69	8.89 (0.34)	254	8.89 (0.32)	8	8.80 (0.31)	0.726	68	9.02 (0.31)	191	9.03 (0.27)	17	8.95 (0.25)	0.500
Vitamin D (nmol/L)	69	41.51 (18.66)	254	45.03 (16.91)	8	39.56 (12.47)	0.242	69	33.44 (16.48)	191	34.53 (14.81)	17	38.38 (18.34)	0.499
Smoking	85		280		8		**0**.**001**	75		199		17		**0**.**002**
No, never	56	65.9%	240	85.7%	8	100.0%		51	68.0%	171	85.9%	14	82.4%	
Sometimes	24	28.2%	34	12.1%	0	0.0%		18	24.0%	27	13.6%	3	17.6%	
Daily	5	5.9%	6	2,1%	0	0.0%		6	8.0%	1	0.5%	0	0%	
Snuffing	85		280		8		0.298	75		198		17		**0**.**010**
No, never	52	61.1%	198	70.7%	7	87.5%		38	50.7%	143	72.2%	11	64.7%	
Sometimes	14	16.5%	40	14.3%	1	12.5%		11	14.7%	21	10.6%	1	5.9%	
Daily	19	22.4%	42	15.0%	0	0%		26	34.7%	34	17.2%	5	29.4%	
Alcohol intake	85		280		8		**0**.**024**	74		199		17		**<0**.**001**
Never	14	16.5%	77	27.5%	4	50.0%		16	21.6%	76	38.2%	9	52.9%	
Once per month or less	37	43.5%	132	47.1%	3	37.5%		27	36.5%	84	42.2%	3	17.6%	
Twice or more per month	34	40.0%	71	25.4%	1	12.5%		34	45.9%	39	19.6%	5	29.4%	
Physical activity	85		280		8		**0**.**015**	75		199		17		0.331
Physically inactive	15	17.6%	32	11.4%	0	0.0%		24	32.0%	52	26.1%	4	23.5%	
Some light physical activity	42	49.4%	103	36.8%	4	50.0%		16	21.3%	51	25.6%	3	17.6%	
Regular physical activity and training	22	25.9%	86	30.1%	2	25.0%		21	28.0%	48	24.1%	2	11.8%	
Regular hard physical activity (for competitive sports)	6	7.1%	59	21.1%	2	25.0%		14	18.7%	48	24.1%	8	47.1%	
Current infection	85		278		8		0.667	75		198		17		0.330
No	74	87.1%	236	84.9%	8	100.0%		64	85.3%	165	83.3%	12	70.6%	
Yes	11	12.9%	42	15.1%	0	0.0%		11	14.7%	33	16.7%	5	29.4%	
Chronic disease	85		278		8		0.725	75		197		17		
No	61	71.8%	187	67.3%	6	75.0%		59	78.7%	141	71.6%	9	52.9%	
Yes	24	28.2%	91	32.7%	2	25.0%		16	21.3%	56	28.4%	8	47.1%	
Hormonal contraceptives	85		277		8		0.083							
No	44	51.8%	179	64.6%	6	75.0%								
Yes	41	48.2%	98	35.4%	2	25.0%								
PDS status								63		158		16		0.358
Not begun								0	0.0%	0	0.0%	0	0.0%	
Barely started								7	11.1%	16	10.1%	0	0.0%	
Underway								48	76.2%	111	70.3%	11	68.8%	
Completed								8	12.7%	31	19.6%	5	31.3%	
High School Program	85		280		8		0.219	75		199		17		**0**.**006**
General Studies	44	51.8%	155	55.4%	4	50.0%		24	32.0%	81	40.7%	2	11.8%	
Sports and Physical	4	4.7%	31	11.1%	0	0.0%		6	8.0%	31	15.6%	6	35.3%	
Vocational	37	43.5%	94	33.6%	4	50.0%		45	60.0%	87	43.7%	9	52.9%	

Bold: Statistically significant with a *p*-value of 0.05. Mean (SD) of continuous variable and percentages of categorical variables are reported. BMI, Body Mass Index; hs-CRP, high-sensitive C-reactive protein; IL6-α: Interleukin 6 alpha; TGF-α: Transforming growth factor alpha; TRANCE, Tumor Necrosis Factor-related activation-induced cytokine (O14788: TNF-related activation-induced cytokine within limits of detection); TWEAK, Tumor necrosis factor-like weak inducer of apoptosis (O43508: TNF-like weak inducer of apoptosis within limits of detection); NPX, Normalized protein expression; Vitamin D, Standardized version of (25-OH)D; PDS status, Pubertal Development Scale status.

*One-way ANOVA was used to compare continuous data across the sleep duration categories. When Levene’s test indicated unequal variances, Welch’s corrected *F*- and *p*-values are reported. Fisher’s exact test was used to compare categorical data across sleep duration.

### Cross-sectional associations between sleep duration and HSCL-10

At baseline, a 30-minute increase in sleep duration (one unit) was associated with a significant lower level of HSCL-10 in girls B [95% CI] = −0.132 [−0.183, −0.081] and boys, B [95% CI] = −0.089 [−0.127, −0.051]. At follow-up, the corresponding crude associations were significant among girls, *B* [95% CI] = −0.134 [−0.186, −0.082] and boys, *B* [95% CI] = −0.087 [−0.132, −0.042] (Table 2).

**Table 2.** Crude and adjusted associations between sleep duration and psychological distress (Hopkins Symptom Check List HSCL-10), assessed by linear regressions, 1 unit sleep duration equals 30 min.

			**95% CI**			
	***n***	***B***	Lower	Upper	***p*-value**	***R*^2^**
**Girls**						
Baseline univariate	373	−0.132	−0.183	−0.081	<0.001*	0.066
Follow-up univariate	372	−0.134	−0.186	−0.082	<0.001*	0.065
Model 1	372	−0.090	−0.131	−0.048	<0.001*	0.047
Model 2	361	−0.068	−0.109	−0.027	0.001*	0.137
**Boys**						
Baseline univariate	291	−0.089	−0.127	−0.051	<0.001*	0.069
Follow-up univariate	294	−0.087	−0.132	−0.042	<0.001*	0.048
Model 1	291	−0.054	−0.091	−0.017	0.005*	0.027
Model 2	117	−0.078	−0.125	−0.031	0.001*	0.376

The results are presented for girls and boys, respectively. Fit Futures from baseline 2010–2011 and follow-up in 2012–2013. *B*: Unstandardized beta. Baseline univariate: Crude analysis baseline. Follow-up univariate: Crude analysis follow-up. Model 1: Crude analysis with change score sleep duration as exposure and change score Hopkins Symptom Checklist (HSCL-10) as outcome. Model 2 for girls: Model 1 + use of contraceptives, change score smoking, change score snuffing, change score physical activity and change score self-rated health. Model 2 for boys: Model 1 + chronic disease, change score current infection, change score self-rated health, change score smoking, high-sensitive C-reactive protein, interleukin 6 alpha and transforming growth factor alpha.

*Statistically significant with a *p*-value cut-off of 0.05.

### Associations between change in sleep duration and change in HSCL-10

In girls, increasing sleep duration by 30 min from baseline to follow-up (one unit) was associated with a significant decrease in HSCL-10 from baseline to follow-up *B* [95% CI] = −0.090 [−0.131, −0.048]. In boys, the corresponding association was also significant, *B* [95% CI] = −0.054 [−0.091, −0.017]. The associations remained significant after adjustment for potential confounders in both girls, *B* [95% CI] = −0.068 [−0.109, −0.027] and boys, *B* [95% CI] = −0.078 [−0.125, −0.031] (Table 2). Only 131 boys had data on current infection at follow-up. This is the main reason for low n (117) in Model 2. Removing the change score of the current infection from Model 2 (*n *= 259) did not change the result (data not shown).

### Supplementary analysis

Removing the sleep item from HSCL-10 did not change the significant cross-sectional findings from neither baseline nor follow-up. Similarly, the significant associations between change in sleep duration and change in HSCL-10 remained significant when excluding the sleep item (Supplementary table 1).

Adjusting for potential confounders from baseline only did not change the results (data not shown).

## Discussion

In this population-based prospective study on adolescents between 15 and 18 years of age, we found significant prospective associations between sleep duration and psychological distress in both girls and boys. Reduced sleep duration was associated with increased psychological distress. Inverse cross-sectional associations between sleep duration and psychological distress were also apparent at baseline as well as at follow-up. With an average sleep duration of 7 h per night, this sample slept less than the recommended 8–10 h. Approximately, 25% of both genders slept less or equal to 6 h, and 1–3% (dependent on time-point and gender) slept above 9 h per night.

After adjusting for confounders, we found that one unit (30 min) increase in sleep duration from baseline to follow-up was associated with a decrease in HSCL-10 score from baseline to follow-up of 0.068 in girls and 0.078 in boys. Previous reports indicate that the association between insomnia and depressive symptoms is stronger in girls compared to boys (Langvik et al., 2019). However, in the present study, the effect sizes (in form of beta-values) were similar across gender. Our findings on prospective associations between change in sleep duration and change in psychological distress support the results of Fuligni et al. (2019). In a study including approximately 750 healthy adolescents 14–15 years of age, they found that short sleep duration was correlated with increased psychological distress the following day. To consider individual differences in the association between sleep duration and psychological distress, Fuligni et al. (2019) used daily measurements for a 2-week period. Studies have reported prospective associations between sleep duration and psychological distress with longer time lags. A study by Orchard et al. (2020) reported that sleep duration on school nights among adolescents 15 years of age predicted psychological distress at the age of 21 (Orchard et al., 2020). Thus, our findings of prospective associations between change in sleep duration and change in psychological distress over two years are consistent with studies both with shorter and longer time lags.

Our finding that an increase of 30 min in sleep from baseline to follow-up was associated with a decrease in psychological distress from baseline to follow-up may be clinically relevant in adolescents that are sleep deprived. In support, two studies that evaluated the effect from delayed school start (Boergers et al., 2014; Chan et al., 2018), and a study that evaluated a sleep education program for adolescents (Bonnar et al., 2014) reported that an increase of approximately 30 min of sleep duration was associated with decreased psychological distress and decreased depressed mood. In their randomized controlled trial (RCT), Bonnar et al. (2014) found that an increase of 27 min of sleep duration in school nights in the intervention group, resulted in a decline in depressed mood compared to the control group.

On the other hand, there are findings pointing towards no prospective associations between sleep duration and symptoms of depression and anxiety. Doane et al. (2015) found no significant prospective associations between sleep duration and symptoms of anxiety and depression, in adolescents 18 years of age at baseline. Nevertheless, their sample size was small (*n *= 71), and the association might have been significant with a bigger sample size. A larger study by Fan et al. (2017) found no association between sleep duration at baseline and depressive symptoms at follow-up one year later in adolescents 15 years of age at baseline. A difference from our study is that Fan et al. (2017) adjusted for baseline depressive symptoms; whereas we, in our study, used change scores of sleep duration and psychological distress as exposure and outcome, respectively. Adjusting for baseline depressive symptoms examines whether sleep duration at baseline is associated with depressive symptoms at follow-up, whereas using change scores examines whether a change in sleep duration from baseline to follow-up is associated with a change in psychological distress from baseline to follow-up. Lovato et al. (2017) suggested that effect from insufficient sleep on depressed mood may be relatively short. Our approach with change scores may be said to consider such short effects from sleep duration by including sleep duration at follow-up in the exposure.

A weakness with measuring sleep duration by one item is that it does not discriminate between sleep duration on weekdays and weekends. This discrimination is important because it has been shown that adolescents sleep 1–2 h longer on weekends compared to weekdays (Carskadon, 2011). Further, we did not have data on whether the participants slept during daytime (napping). Adolescents with short sleep duration during the night have been reported to nap during the day (Santos et al., 2021). However, other studies (Lee & Sibley, 2019; Marques et al., 2021) have used the following single item from the Pittsburgh Sleep Quality Index to measure sleep duration: ‘During the past month, on average, how many hours of actual sleep did you get per night?' This item is comparable to the item we used. Further, our descriptive findings on sleep duration in adolescents correspond to findings from studies that used questionnaires, actigraphy and interviews to measure sleep duration (Gradisar et al., 2011; Roberts et al., 2011), indicating that our measure is valid for describing sleep duration. Finally, our aim was merely to explore (not examine) prospective associations between sleep duration and psychological distress.

Our findings on mean sleep duration of approximately 7 h are in line with a review and meta-analysis on adolescents from Europe, America and Asian countries (Gradisar et al., 2011), where mean sleep duration was found to be approximately 7 h in participants 15–18 years of age. Our findings also correspond with a study from Norway, on 4010 first-years high school students, aged 16–17 years, who reported a mean sleep duration of 6 h and 43 min on school nights (Saxvig et al., 2021). A study on 4175 American adolescents aged 11–17 years at baseline reported that 20% slept ≤6 h at baseline and 17% slept below 6 h at follow-up (Roberts et al., 2011). These percentages are somewhat below the percentages that slept ≤6 h in our study; however, the sample in the referenced study is younger than the sample in our study.

This study has several strengths. The participants were healthy adolescents, and the attendance rate was very high. Adolescents are understudied in general and with respect to the prospective association between sleep duration and psychological distress. All analyses were performed gender-stratified since earlier studies have shown gender-differences in prevalence of both psychological distress and associations between sleep duration and depressive symptoms. Our study examined several potential confounders. Finally, we explored associations between change in sleep duration and change in psychological distress. This approach considers individual differences in sleep need, which has been recommended (Fuligni et al., 2019), and explores whether sleep duration and psychological distress vary together across time.

This is an observational study, and we cannot exclude residual confounding. There may be other factors that have caused changes in both sleep duration and psychological distress. Stratification on gender reduces statistical power. However, our sample sizes were big enough to detect significant associations in both genders, in crude and adjusted analyses. The beta-values are relatively small, and the clinical relevance may be questioned. On the other side, small effect-sized are common in psychological research (Funder & Ozer, 2019). Our assumption that changes in sleep duration represent the same exposure across the scale may be questioned. For example, a change in sleep duration from 6.0 to 6.5 h, may be different from a change from 8.0 to 8.5 h. The association between change in sleep duration and change in psychological distress may be dependent upon sleep duration level at baseline. There will probably be an upper threshold at which additional sleep will not be associated with lower levels in psychological distress. Most of the adolescents slept below the recommended hours, and thus it is likely that increasing sleep duration with 30 min may be beneficial for most of the participants. There are individual differences in sleep need (Meltzer, 2017), hence it may be that change in sleep duration is the sleep variable most strongly associated with change in psychological distress.

## Conclusion

In this study, short sleep duration was highly prevalent in healthy adolescent girls and boys. Increased sleep duration from baseline to follow-up was associated with decreased psychological distress from baseline to follow-up, in both genders, and vice versa, decreased sleep duration was associated with increased psychological distress. Due to variability in the results of the existing research, there is a need for more studies examining associations between sleep duration and psychological distress.

## Supplementary Material

Supplemental Material

## Data Availability

Data may be obtained from a third party, UiT – The Artic University of Norway. Restrictions apply to the availability of these data, which were used under license for the current study, and are thus not publicly available.
