# Supplementary material for: Two-year changes in sleep duration are associated with changes in psychological distress in adolescent girls and boys: the fit futures study
Source: Health Psychol Behav Med. 2022 Nov 21;10(1):1159–75. doi: 10.1080/21642850.2022.2147936 (PMC9683068; doi:10.1080/21642850.2022.2147936)
Supplement: Supplemental Material [file RHPB_A_2147936_SM8943.docx]

**Supplementary Table 1:** *Crude and adjusted* *associations between sleep duration (1 unit = 30 minutes) and Hopkins Symptom Check List (HSCL-10) without the sleep item, assessed by linear regression*. *The results are presented for girls and boys, respectively.* *Fit Futures baseline in 2010-2011 and follow-up in 2012-2013.*

| Girls |  | |  | |  | | | | |
| --- | --- | --- | --- | --- | --- | --- | --- | --- | --- |
|  | | | | 95 % CI | | |  |  |  |
|  | *n* | *B* | | Lower | | Upper | *p*-value | R^2^ |  |
| Baseline univariate | 373 | -0.122 | | -0.173 | | -0.071 | <0.001* | 0.057 |  |
| Follow-up univariate | 372 | -0.120 | | -0.173 | | -0.067 | <0.001* | 0.051 |  |
| Model 1 | 372 | -0.062 | | -0.099 | | -0.024 | 0.001* | 0.028 |  |
| Model 2 | 361 | -0.043 | | -0.080 | | -0.006 | 0.025* | 0.120 |  |
| Boys |  |  | |  | |  |  |  |  |
|  | | | | 95 % CI | | |  |  |  |
|  | *n* | *B* | | Lower | | Upper | *p*-value | R^2^ |  |
| Baseline univariate | 291 | -0.075 | | -0.113 | | -0.038 | <0.001* | 0.053 |  |
| Follow-up univariate | 294 | -0.076 | | -0.121 | | -0.031 | 0.001* | 0.036 |  |
| Model 1 | 291 | -0.042 | | -0.074 | | -0.010 | 0.010* | 0.023 |  |
| Model 2 | 117 | -0.067 | | -0.109 | | -0.025 | 0.002* | 0.184 |  |

*B*: Unstandardized beta

*Statistically significant with a p-value of 0.05

Baseline univariate: Crude analysis baseline

Follow-up univariate: Crude analysis follow-up

Model 1: Crude analysis with change score sleep duration as exposure and change score Hopkins Symptom Checklist (HSCL-10) without the sleep item as outcome

Model 2 for girls: Model 1 + use of contraceptives, change score smoking, change score snuffing, change score physical activity and change score self-rated health

Model 2 for boys: Model 1 + chronic disease, change score current infection, change score self-rated health, change score smoking, high-sensitive C-reactive protein, interleukin 6 alpha and transforming growth factor alpha
